# QTL mapping of agronomic traits in wheat using the UK Avalon ×  Cadenza reference mapping population grown in Kazakhstan

**DOI:** 10.7717/peerj.10733

**Published:** 2021-02-18

**Authors:** Akerke Amalova, Saule Abugalieva, Vladimir Chudinov, Grigoriy Sereda, Laura Tokhetova, Alima Abdikhalyk, Yerlan Turuspekov

**Affiliations:** 1 Institute of Plant Biology and Biotechnology, Almaty, Kazakhstan; 2 Faculty of Biology and Biotechnology, Al-Farabi Kazakh National University, Almaty, Kazakhstan; 3 Karabalyk Agricultural Experimental Station, Nauchnoe, Kostanai Region, Kazakhstan; 4 Karaganda Research Institute of Agriculture, Karaganda, Kazakhstan; 5 Kazakh Rice Research Institute, Kyzylorda, Kazakhstan; 6 Faculty of Agrobiology, Kazakh National Agrarian University, Almaty, Kazakhstan

**Keywords:** Bread wheat, Doubled haploid population, Quantitative trait loci, Genetic map, DNA markers, Marker-trait associations

## Abstract

**Background:**

The success of wheat production is largely dependent on local breeding projects that focus on the development of high-yielding cultivars with the use of novel molecular tools. One strategy for improving wheat productivity involves the deployment of diverse germplasms with a high potential yield. An important factor for achieving success involves the dissection of quantitative trait loci (QTLs) for complex agronomic traits, such as grain yield components, in targeted environments for wheat growth.

**Methods:**

In this study, we tested the United Kingdom (UK) spring set of the doubled haploid (DH) reference population derived from the cross between two British cultivars, Avalon (winter wheat) and Cadenza (spring wheat), in the Northern, Central, and Southern regions (Karabalyk, Karaganda, Kyzylorda) of Kazakhstan over three years (2013–2015). The DH population has previously been genotyped by UK scientists using 3647 polymorphic DNA markers. The list of tested traits includes the heading time, seed maturation time, plant height, spike length, productive tillering, number of kernels per spike, number of kernels per meter, thousand kernel weight, and yield per square meter. Windows QTL Cartographer was applied for QTL mapping using the composite interval mapping method.

**Results:**

In total, 83 out of 232 QTLs were identified as stable QTLs from at least two environments. A literature survey suggests that 40 QTLs had previously been reported elsewhere, indicating that this study identified 43 QTLs that are presumably novel marker-trait associations (MTA) for these environments. Hence, the phenotyping of the DH population in new environments led to the discovery of novel MTAs. The identified SNP markers associated with agronomic traits in the DH population could be successfully used in local Kazakh breeding projects for the improvement of wheat productivity.

## Introduction

Hexaploid wheat (*Triticum aestivum* ssp. *aestivum* L. em. Thell.) is one of the most abundant sources of energy and proteins for the world’s population. Bread wheat genome is hexaploid, and consists of three-component genomes—A, B, and D, each comprising seven chromosomes—share many regions of high similarity (International Wheat Genome Sequencing Consortium, 2018). Genome size estimated at ∼17 Gb. The ancestral progenitor genomes are considered to be *Triticum urartu* (the A-genome donor), *Aegilops speltoides* (the B-genome donor). This first hybridisation event produced tetraploid emmer wheat (AABB, *T. dicoccoides*) which hybridized again with *Aegilops tauschii* (the D-genome donor) to produce modern bread wheat (Ensembl Plants, 2020). Its increased production is essential for food security on a global scale (*Curtis & Halford, 2014*). Wheat occupies approximately 17% of the total cropland and contributes around 35% of the staple food in many countries (*Mitikul & Regassa, 2019*). In Kazakhstan, spring wheat is a leading crop due to the favorable agroclimatic conditions, and our country currently amongst the top ten bread wheat producers in the world and a major exporter. However, the average yield of wheat in Kazakhstan is only 1.2 tons per hectare (ha) (USDA, 2018), while the Food and Agriculture Organization of the United Nations (FAO) predicts that the country could potentially increase productivity up to 3 tons/ha (Alexandratos & Bruinsma, 2012).

To meet this target, several requirements need to be met first, including improvements in agronomy, better prediction of the changing climate across Kazakhstan (which is the ninth largest country in the world), and the breeding of new cultivars with high productivity and quality. In this study, we hope to contribute to meeting the last of these requirements. The development of competitive cultivars requires focused projects that should incorporate extensive germplasm evaluation as well as modern genetics and breeding tools, with the aim of introducing new and novel genetic variations. As wheat agronomic traits show continuous variation and are controlled by many genes, the analysis of quantitative trait loci (here, QTL for single and QTLs for plural) is of great importance for modern plant breeding.

During the last few decades, many QTL mapping studies in wheat have been performed in different parts of the world (*Jantasuriyarat et al., 2004; Lobell et al., 2005; Cuthbert et al., 2008; Heidari et al., 2011; Cavanagh et al., 2013; Echeverry-Solarte et al., 2015*) providing a robust platform for the improvement of breeding efficiency via the successful introduction of marker-assisted selection (*Kuchel et al., 2005; Gupta, Langridge & Mir, 2010; Lopes et al., 2015*) and genomic breeding approaches (*Jannink, Lorenz & Iwata, 2010; Heffner, Jannink & Sorrells, 2011; Poland et al., 2012*).

Despite the recent sharp rise in the importance of genome-wide association studies (GWAS) (Sukumaran et al., 2015; Zanke et al., 2015) in wheat, including those performed in Kazakhstan (*Turuspekov et al., 2017; Anuarbek et al., 2020; Genievskaya et al., 2020*), QTL analyses based on the use of biparental mapping populations and associated linkage maps still play an important role in the genetic dissection of complex traits associated with yield and its components (*Cuthbert et al., 2008; Van Eeuwijk et al., 2010; Zhou et al., 2017; El-Feki et al., 2018; Onyemaobi et al., 2018; Tura et al., 2020*). The importance of this approach relies on the rapid construction of an appropriate mapping population (MP), an abundance of recombination, good phenotyping capability, and the availability of automated single nucleotide polymorphism (SNP) genotyping platforms.

Biparental MPs were successfully used in studies of abiotic (*Roy, Tucker & Tester, 2011; Bansal, Lenka & Mondal, 2014; Sehgal, Baliyan & Kaur, 2019*) and biotic stress tolerances (*Bennett et al., 2012*), and grain quality (*Abugalieva et al., 2010; Smith et al., 2011; Abugalieva et al., 2014; Goel et al., 2019*). Among the different types of biparental populations, doubled haploid (DH) populations are often used in a family-based mapping approach (*Xu et al., 2017*) as this instantly eliminates the issue of heterozygosity within the studied lines. There are many examples where DH mapping populations have been used successfully for the construction of genetic maps of hexaploid wheat and QTL mapping (*Blake et al., 2019* ). One example of the prominent use of DH lines in the identification of marker-trait associations in the UK is by having a national reference population, in this case, Avalon × Cadenza (A × C) (*Griffiths et al., 2009; Griffiths et al., 2012; Allen et al., 2011; Bai, Liang & Hawkesford, 2013; Ma et al., 2015; Farré et al., 2016; Coulton et al., 2020; Thirkell, Pastok & Field, 2020*), which was developed as part of the UK Wheat Genetic Improvement Network (WGIN, 2008) and tested for agronomic traits in different regions the world (*Ma et al., 2015; Farré et al., 2016*),

Previously, a Chinese Spring × SQ1 doubled haploid mapping populations developed in the UK was successfully tested in the Southeast (SE) of Kazakhstan (*Quarrie et al., 2005; Abugalieva, 2007*). The results of the study suggest that the MP constructed in the UK was well suited for plant growth in SE Kazakhstan (*Quarrie et al., 2005; Abugalieva, 2007; Abugalieva et al., 2010; Abugalieva et al., 2014*). In this work, it was assumed that 101 spring DH lines of the A × C would also be well adapted to the different conditions of Kazakhstan, and for the first time, it was studied in conditions of Kazakhstan. Therefore, the purpose of this study was to identify QTLs for key agronomic traits using the UK reference MP A × C tested in three wheat-growing regions of Kazakhstan during three years of trials, 2013–2015. The experiments were conducted within the international “ADAPTAWHEAT” project supported by 7th Framework programme of the European Union (ADAPTAWHEAT, 2012).

## Materials & Methods

### Avalon × Cadenza mapping population

The original mapping population (MP) Avalon × Cadenza (A × C) consisted of 201 samples including 100 winter type lines and 101 spring type lines The MP was produced from a cross between widely grown British wheat cultivars Avalon (winter wheat) and Cadenza (spring wheat). The A × C DH population was developed as part of the Wheat Genetic Improvements Network (WCIN) (http://www.wgin.org.uk/) (*Allen et al., 2011*). The parental cultivars differ in their photoperiod sensitivity alleles by: *Ppd-A1, Ppd-D1*, *Ppd-B1*, and vernalization genes *Vrn-A1, Vrn-B1, Vrn-D1* (Avalon) and *Vrn-A1a* (Cadenza). They also differ in terms of reduced height genes as Avalon carries the allele *Rht-D1b*, while Cadenza carries the wild type allele *Rht-D1a* (*Farré et al., 2016*). In this study, only spring-type DH lines and Cadenza were subjected for the analysis along with local standards.

### Evaluation of the MP for variation in agronomic traits

The studied traits were formally divided into two groups: plant adaptation-related traits and yield components. The plant adaptation traits included the heading time (HT, days), seed maturation time (SMT, days), and plant height (PH, cm). The yield components, including the spike length (SL, cm), productive tillering (PT, pcs), number of kernels per spike (NKS, pcs), thousand kernel weight (TKW, g), and number of kernels per meter (NKM, pcs), were calculated as PT × NKS, yield per square meter (YM2, g). These A × C spring lines were evaluated in three regions of Kazakhstan, at the Karabalyk Agricultural Experimental Station (North Kazakhstan), the Karaganda Institute of Agriculture (Central Kazakhstan), and the Kazakh Rice Research Institute (South Kazakhstan) over three years, 2013–2015 (Fig. S1). In Northern and Central Kazakhstan, DH lines were grown in non-irrigated plots, while in Southern Kazakhstan, plants were grown in an irrigated field. DH lines and Cadenza were planted in three replications at each location in randomized 1 m^2^ plots. In addition, local standards “Karabalykskaya 90”, “Karagandinskaya 22”, and “Kazakhstanskaya 4”, were planted in Northern, Central, and Southern Kazakhstan, respectively. The distance between rows was 15 cm, and the distance between plants in a row was 5 cm, respectively (*Dospekhov, 1985*). The climate conditions recorded during the trials were shown in Table 1, and more extended climate information for the last eight years was provided in the Raw data file.

**Table 1:** Location, environment, and weather data at three breeding stations in Kazakhstan where the Avalon × Cadenza mapping population was grown.

Site/Region	**KB (North)**			**KA (Center)**			**KO (South)**		
Latitude/Longitude	53.45/62.03			49.40/72.41			44.51/65.30		
Altitude, m	189			570			129		
Soil type	Black soil (humus 4.5–5.0%)			Dark chestnut (humus 3.0–3.5%)			Meadow-marsh (humus 1.97–1.98%)		
Year	2013	2014	2015	2013	2014	2015	2013	2014	2015
Average Rainfall, mm	62.3			32.6			11.6		
Annual rainfall, mm	88.9	54.14	43.7	26.3	33.0	38.5	9.2	8.8	17.0
Mean temperature, °C	17.4	18.3	17.9	15.4	17.7	16.3	23.1	22.4	23.9
Max temperature, °C	22.1	23.1	24.6	18.6	20.1	20.3	28.6	28.7	29.7
Min temperature, °C	12.2	11.8	13.6	11.4	14.0	10.4	15.2	10.6	15.1
Conditions*	Rainfed			Rainfed			Irrigated		

**Notes.**

KB Karabalyk Agricultural Experimental Station KA Karaganda Institute of Agriculture KO Kazakh Rice Research Institute

### Linkage mapping and QTL analysis

The genetic map for A × C was developed by Griffiths et al. (2009) and Griffiths et al. (2012), and details of this map are available from the WGIN website (WGIN, 2008). The genetic map was previously reported to consist of 3647 polymorphic DNA markers, including 22 amplified fragment length polymorphisms (AFLPs), 16 COS (conserved orthologous sequences), 88 DArT (diversity array technology), 3325 SNPs (single-nucleotide polymorphisms), 153 SSRs (simple sequence repeats), 3 STSs (sequence-tagged sites), and 12 genes (Table S1). The total map length was 3246.9 centiMorgan (cM), with an average chromosome length of 154.6 cM; range: 16.80 cM (chromosome 6D) to 264.8 cM (chromosome 5B) (*Griffiths et al., 2009; Griffiths et al., 2012*). QTL identification was conducted using the composite interval mapping (CIM) methods of Windows QTL Cartographer v2.5 software (*Wang, Basten & Zeng, 2012*). A logarithm of the odds ratio (LOD) threshold of 3.0 was used to determine a significant QTL. MapChart v2.32 software was used to draw the genetic map (*Voorrips, 2002*). The correlation analysis was calculated using the Rstudio software (R Studio Team, 2020). The GGE (genotype plus genotype-by-environment interaction) effects were analyzed using GenStat software (International, 2019).

## Results

### Evaluation of agronomic traits of the A ×C population in three regions of Kazakhstan

The duration of HT differed sharply across the three regions based on the analysis of means over three years (Table 2). The earliest HT was registered in the Karaganda Institute of Agriculture (KA) region (42.3 ± 1.11 days), followed by Karabalyk Agricultural Experimental Station (KB) (49.4 ± 1.32 days), and then Kazakh Rice Research Institute (KO) (56.6 ± 3.09 days). The duration of the mean SMT showed a different trend for the three regions, with the earliest seed maturation observed in KO (22.5 ± 2.18 days), followed by KA (47.2 ± 1.16 days), and then KB (49.0 ± 1.35 days). The mean PH ranged from 47.1 ± 5.6 cm in KA to 58.1 ± 5.9 cm in KO (Table 2).

**Table 2:** The means for agronomic traits in the Avalon × Cadenza mapping population (2013–2015).

**Region**	**Traits**	**HT, days**	**SMT, days**	**PH, cm**	**SL, cm**	**PT, pcs**	**NKS, pcs**	**TKW, g**	**NKM, pcs**	**YM2, g**
**KB**	DHL (min)	45.5	45.5	32.9	4.7	1.5	10.7	30.0	15.2	188.2
	DHL (max)	52.0	52.3	64.1	7.1	2.9	21.7	36.1	56.0	847.2
	DHL (mean ± SD)	49.4 ± 1.3	49.0 ± 1.4	53.4 ± 5.2	6.05 ± 0.5	2.3 ± 0.3	14.6 ± 2.8	33.3 ± 1.4	26.3 ± 7.1	402.1 ± 113.2
	**Cadenza**	**49.0**	**46.0**	**53.2**	**6.23**	**2.2**	**13.4**	**32.1**	**37.1**	**466.6**
	**Karabalykskaya 90**	**40.3**	**46.3**	**54.6**	**5.96**	**2.3**	**16.2**	**33.8**	**37.5**	**556.7**
**KA**	DHL (min)	39.6	44.7	36.4	4.1	1.2	19.0	28.9	21.9	61.5
	DHL (max)	44.6	49.3	60.7	8.0	1.6	34.9	38.7	53.2	450.2
	DHL (mean+SD)	42.3 ± 1.1	47.2 ± 1.2	47.1 ± 5.6	6.37 ± 0.6	1.3 ± 0.1	26.5 ± 3.3	33.6 ± 2.4	33.9 ± 5.7	146.5 ± 58.2
	**Cadenza**	**42.7**	**50.0**	**44.3**	**6.1**	**1.1**	**27.8**	**30.0**	**32.3**	**101.2**
	**Karagandinskaya 22**	**38.3**	**50.3**	**51.2**	**5.7**	**1.3**	**24.5**	**45.8**	**30.6**	**163.5**
**KO**	DHL (min)	50.6	10.3	46.0	6.9	1.5	40.5	33.2	66.4	241.4
	DHL (max)	64.0	27.6	71.4	9.7	2.7	49.7	41.6	119.3	564.2
	DHL(mean+SD)	56.6 ± 3.09	22.5 ± 2.2	58.1 ± 5.9	8.35 ± 0.6	2.01 ± 0.3	45.4 ± 1.9	37.8 ± 1.7	91.9 ± 10.6	432.3 ± 57.9
	**Cadenza**	**51.3**	**26.3**	**62.0**	**8.2**	**2.6**	**43.6**	**39.3**	**112.9**	**540.3**
	**Kazakhstanskaya 4**	**56.3**	**25.6**	**85.7**	**8.76**	**2.0**	**44.2**	**35.3**	**104.2**	**466.7**

**Notes.**

DHL Double Haploid line KB Karabalyk Agricultural Experimental Station KA Karaganda Institute of Agriculture KO Kazakh Rice Research Institute

As the KB station represents Northern Kazakhstan, where wheat is grown on over 80% of the total sowing area in Kazakhstan, it was essential to compare the MP to the local standard (check cultivar) “Karabalykskaya 90”. The comparison showed that the mean performance for the HT, SMT, and PH in the A × C lines was less optimal than for the local standard. Notably, the average HT was 9.1 days, and the average SMT was 2.7 days longer in comparison with Karabalykskaya 90. This pattern was also observed at KA (Central Kazakhstan), but was reversed at the KO station (Southern Kazakhstan) as in the latter case; the SMT was shorter than in the check cultivar for south Kazakhstan, “Kazakhstanskaya 4” (Table 2). Under the irrigated conditions of Southern Kazakhstan (KO), the mean yield of the A × C lines was comparable with the check cultivar, and Cadenza showed even better productivity in comparison to Kazakhstanskaya 4 (Table 2).

An analysis of the means for YM2 revealed that nine DH lines exceeded the YM2 of the local standard cultivar, Karabalykskaya 90 (556.7 g/m^2^) in Northern Kazakhstan. Similar calculations performed for the trials in the Central and Southern regions suggested that 22 and 26 DH lines, respectively, had heavier yields than the corresponding local standard cultivars. Two particular lines, A × C52 and A × C55, demonstrated higher productivity than the check cultivars in all three regions. The averaged YM2 over three years in non-irrigated sites of KB and KO were significantly correlated (*P* < 0.01), and the averages in both locations were not correlated with the irrigated sites in KO (*P* < 0.81).

**Figure 1:**
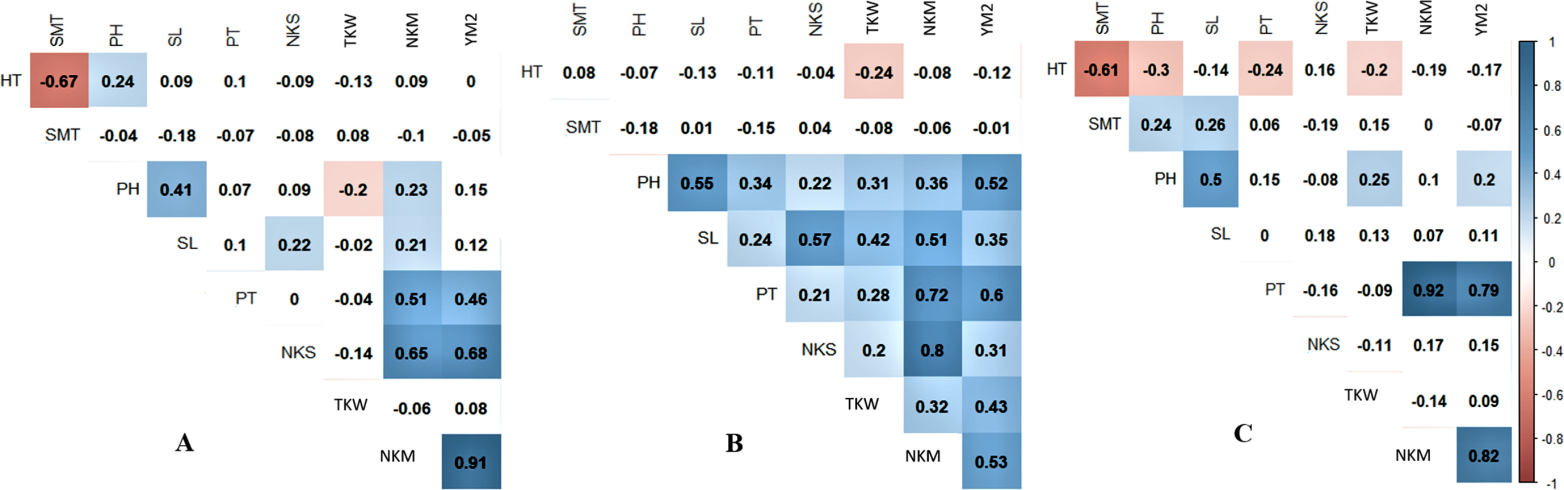
Pearson’s correlation index among means of studied over three years (2013–2015) in three regions of Kazakhstan, Northern (A), Central (B), and Southern (C) stations of studied samples. Correlations with *P* < 0.05 are highlighted in color. The color indicates either positive (blue) or negative (red) correlation.

Pearson’s correlation index showed that in Northern Kazakhstan, the yield was not correlated with HT, SMT, and PH (Fig. 1A). However, TKW, which is one of the important agronomic traits, was negatively correlated with PH (*P* < 0.05), suggesting that plant height is favorable for wheat productivity in this region. A negative correlation of TKW with both HT and SMT and positively correlated with PH was recorded at the KA station, and a negative correlation was revealed between YM2 and SMT at the KO station (Figs. 1B, 1C).

**Figure 2:**
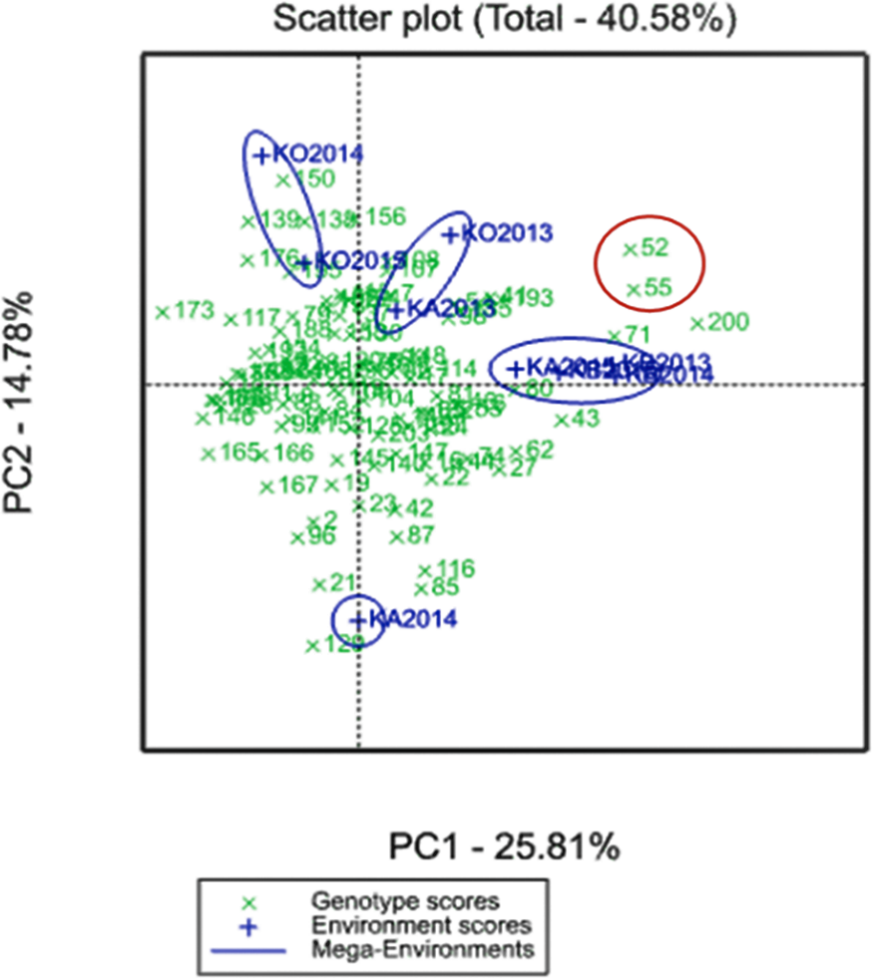
GGE biplot for the averaged YM2 (yield per square meter) over three years (2013–2015) in the Northern (KB, Karabalyk), Central (KA, Karaganda), and Southern (KO, Kyzlorda) regions. Regions are shown in blue, and genotypes (Avalon × Cadenza doubled haploid lines) in green.

A GGE biplot into YM2 divided the three regions for the four mega-environments. PC1 (25.81%) effectively separated KO2014 and KO2015 from the KB and KA sites, and PC2 (14.78%) separated KA2014 from the remaining environments (Fig. 2).

Two particular lines, A × C52 and A × C55, demonstrated adaptability to both non-irrigated and irrigated sites and showed higher productivity than the check cultivars in all three regions located in a biplot between two mega-environments (Fig. 2).

### Identification of QTLs for agronomic traits in three regions of Kazakhstan

The QTL analysis in three studied regions led to the identification of 232 QTLs for nine agronomic traits. The number of QTLs per trait varied from 17 for PH to 40 for NKS (Table 3). Only 83 out of the 232 QTLs found were statistically significantly associated in two and more environments, suggesting that only 1/3 of associations were stable in the three regions. Among the nine traits, the number of identified QTLs varied from two for SMT to 12 each for both PH and SL. The largest LOD score 25.3 for the traits was recorded for PT on chromosome 2D for the Central and Southern regions. The numbers of QTLs among the three genomes A, B, and D were 31, 21, and 27, respectively, suggesting that the A and D genomes were the main locations of the stable associations. The number of stable QTLs identified for the group of adaptation-related traits was eighteen, and the number of QTLs for the group of traits for yield components was seventy-seven (Tables 3 and 4).

**Table 3:** Number of identified in the Avalon × Cadenza mapping population in the three regions (2013–2015 years).

**Trait**	**Total QTL**	**Stable QTL**	**KB**	**KA**	**KO**
HT	18	4	4	1	3
SMT	18	2	2	0	1
PH	17	12	5	5	8
SL	23	12	9	5	9
PT	33	11	9	5	4
NKS	40	11	10	3	5
TKW	33	9	3	4	7
NKM	26	11	5	6	4
YM2	24	11	3	8	4
**Total**	**232**	**83**	**50**	**37**	**45**

**Notes.**

KB Karabalyk Agricultural Experimental Station KA Karaganda Institute of Agriculture KO Kazakh Rice Research Institute

**Table 4:** List of stable QTLs identified in the Avalon × Cadenza mapping population in the three regions (2013-2014-2015 years).

No	**Trait**	**QTL**	**Region**	**Chr**	**Interval cM**	**Reference genome, bp**	**max. LOD score**	**max. R2. %**	**Add. Effect**	**Source of allele increasing trait value**
1	HT	*QHt-AxC.ippb-1D*	KB13-15/KBav	1D	70.8–114.3	462203545-487957083	5.1	17	0.73 day/ −0.73 day	Avalon Cadenza
2	HT	*QHt-AxC.ippb-2D*	KO13, KOav	2D	69.0–92.2	75389775-64733097	5.7	16	1.73 day	Avalon
3	HT	*QHt-AxC.ippb-5B*	KO15/KBav	5B	7.0–43.6	4862138-21538493	5.8	16	1.79/ day −0.42 day	Avalon/ Cadenza
4	HT	*QHt-AxC.ippb-6A*	KO15,KOav, KA14, KAav	6A	30.3–92.3	13806421-425277721	6.1	16	−0.53 day	Cadenza
5	SMT	*QSmt-AxC.ippb-1A*	KB14, KBav	1A	0.0–14.3	4048399-10067369	5.1	16	−0.94 day	Cadenza
6	SMT	*QSmt-AxC.ippb-5A*	KB15, KOav	5A	0.0–32.5	414167574-464478676	4.4	15	−1.21 day	Cadenza
7	PH	*QPh-AxC.ipbb-2A*	KB13, KB14, KO13	2A	72.0–107.6	7853169-31177472	5.2	22	2.74 cm	Avalon
8	PH	*QPh-AxC.ipbb-2B*	KB13, KB14	2B	0.0–43.5	6263398-40905185	4	14	2.25 cm −2.41 cm	Avalon Cadenza
9	PH	*QPh-AxC.ipbb-2D*	KA14, KO13, KO14, KO15	2D	13.1–51.0	13677182-68733980	11.6	19	−2.66 cm	Cadenza
10	PH	*QPh-AxC.ipbb-3A*	KA14, KO14, KO15	3A	49.4–98.5	61343099-680749623	4.3	10	−1.88 cm	Cadenza
11	PH	*QPh-AxC.ipbb-3B*	KA14	3B	62.7–116.4	10202058-38861833	4.9	16	2.48 cm −2.14 cm	Avalon Cadenza
12	PH	*QPh-AxC.ipbb-3D*	KO15	3D	47.4–88.4	552953735-588315426	4.8	13	2.43 cm	Avalon
13	PH	*QPh-AxC.ipbb-4D*	KO13, KO14, KO15	4D	19.9–65.1	3612555-455343893	21.5	48	−4.67 cm	Cadenza
14	PH	*QPh-AxC.ipbb-5A.1*	KB13, KOav	5A	8.3–40.4	30410831-485373904	3.8	13	−2.25 cm	Cadenza
15	PH	*QPh-AxC.ipbb-5A.2*	KO14, KB15	5A	50.5–81.5	533072078-559505885	3.4	16	2.29 cm	Avalon
16	PH	*QPh-AxC.ipbb-5A.3*	KB15/KA13	5A	135.2–183.7	671551553- 706429491	4.8	15	−3.5 cm 3.24 cm	Cadenza Avalon
17	PH	*QPh-AxC.ipbb-5B*	KO14, KO15, Koav	5B	42.7–133.2	568781660-580840106	4.9	13	−4.11 cm	Cadenza
18	PH	*QPh-AxC.ipbb-6B*	KA15, KOav	6B	111.1–136.0	710149821-718232019	4.4	16	−5.14 cm	Cadenza
19	SL	*QSl-AxC.ipbb-1B*	KA15/KO13	1B	7.2–47.1	1523241-59601326	3.9	13	−0.38 cm 0.3 cm	Cadenza Avalon
20	SL	*QSl-AxC.ipbb-2A*	KB15/KO14	2A	93.4–107.3	18234287-31086357	4.5	17	−0.38 cm 0.39 cm	Cadenza Avalon
21	SL	*QSl-AxC.ipbb-2D.1*	KB15/KO13-15,av	2D	24.1–51.0	26774531-68733980	15.7	53	−0.81 cm 0.27 cm	Cadenza Avalon
22	SL	*QSl-AxC.ipbb-2D.2*	KA14, KO14	2D	86.9–193.3	450999021-70951376	3.6	16	−0.54 cm	Cadenza
23	SL	*QSl-AxC.ipbb-3D*	KB15, KA14, KO15	3D	40.2–98.2	524870429-596923394	4.2	13	0.74 cm	Avalon
24	SL	*QSl-AxC.ipbb-4A*	KB13, KBav	4A	104.7–121.6	693278272-715108234	3.9	12	0.22 cm	Avalon
25	SL	*QSl-AxC.ipbb-5A.1*	KB13/KO13	5A	35.3–63.5	473316464-548626258	4.4	17	−0.79 cm 0.23 cm	Cadenza Avalon
26	SL	*QSl-AxC.ipbb-5A.2*	KB14/KO13	5A	91.6–123.7	585430959-659457537	4.5	15	0.85 cm −0.26 cm	Avalon Cadenza
27	SL	*QSl-AxC.ipbb-5A.3*	KB13/KO14	5A	159.5–177.0	689609431-706429491	4.3	16	0.5 cm −0.24 cm	Avalon Cadenza
28	SL	*QSl-AxC.ipbb-5B*	KB13, KO14	5B	86.3–122.4	421275862-551805235	4.5	13	−0.38 cm	Cadenza
29	SL	*QSl-AxC.ipbb-6A*	KB15/KA14	6A	64.1–101.2	51409554-531522308	3.6	11	−0.5 cm 0.67 cm	Cadenza Avalon
30	SL	*QSl-AxC.ipbb-7A*	KA14, KOav	7A	116.5–121.1	515199355-634962318	3.7	13	0.33 cm	Avalon
31	PT	*QPt-AxC.ipbb-1A*	KB13, KB14	1A	39.2–55.4	21760110 -48692389	5.1	17	−0.18 pcs	Cadenza
32	PT	*QPt-AxC.ipbb-1D*	KO14/KB13	1D	75.2–125.0	462203545-556487416	9.0	47	0.41 pcs −0.27 pcs	Avalon Cadenza
33	PT	*QPt-AxC.ipbb-2B*	KA14,15/KB14,15	2B	79.0–87.2	180543407-680409075	6.0	23	0.51 pcs −0.23 pcs	Avalon Cadenza
34	PT	*QPt-AxC.ipbb-2D*	KA14, KO13	2D	67.2–78.9	68733980-123100805	25.3	66	−0.38 pcs	Cadenza
35	PT	*QPt-AxC.ipbb-3D.1*	KA13/KB13,14	3D	22.0–40.4	64767582-524870429	4.3	17	−0.32 pcs 0.10 pcs	Cadenza Avalon
36	PT	*QPt-AxC.ipbb-3D.2*	KB13, KB14	3D	47.5–72.2	552953735-574238844	6.3	18	0.32 pcs	Avalon
37	PT	*QPt-AxC.ipbb-3D.3*	KB13, KB14	3D	91.8–115.0	588315426-613706986	5.3	17	−0.32 pcs	Cadenza
38	PT	*QPt-AxC.ipbb-4B*	KB13, KO14, KO15	4B	98.2–109.3	656816117-656163152	4.8	17	−0.29 pcs	Cadenza
39	PT	*QPt-AxC.ipbb-5A*	KA14, KO14	5A	98.1–105.7	613011972-615863922	23.9	38	−0.29 pcs	Cadenza
40	PT	*QPt-AxC.ipbb-5D*	KA13/KB15	5D	4.3–16.3	3609859-8746800	6.6	22	−0.15 pcs 0.14 pcs	Cadenza Avalon
41	PT	*QPt-AxC.ipbb-7A*	KB13, KB14, KB15, KBav	7A	191.8–212.8	708246600-724085134	6.5	19	−0.3 pcs	Cadenza
42	NKS	*QNks-AxC.ipbb-1A*	KB13, KB15	1A	16.3–31.2	10067369-14046238	4.2	10	0.96 pcs	Avalon
43	NKS	*QNks-AxC.ipbb-2B*	KO14, KB13, KB14	2B	82.1–93.5	641877699-654510653	5.1	13	−3.83 pcs	Cadenza
44	NKS	*QNks-AxC.ipbb-2D.1*	KA13, KB15	2D	11.8–20.6	13677182-13989187	4.0	10	−2.19 pcs	Cadenza
45	NKS	*QNks-AxC.ipbb-2D.2*	KO14, KB15	2D	86.8–107.2	450999021-70951376	3.8	14	−1.41 pcs	Cadenza
46	NKS	*QNks-AxC.ipbb-3D*	KB13, KB14	3D	51.5–72.1	552953735-574238844	3.3	9	1.38 pcs	Avalon
47	NKS	*QNks-AxC.ipbb-4B*	KOav/KB15	4B	47.5–62.3	35728213-535085299	5.8	17	0.83 pcs −2.43 pcs	Avalon Cadenza
48	NKS	*QNks-AxC.ipbb-4D*	KB15	4D	59.6–94.4	32347333-455253024	3.8	10	1.12 pcs	Avalon
49	NKS	*QNks-AxC.ipbb-5A*	KB14, KB15, KBav	5A	21.6–32.1	448109881-465294775	7.6	22	−1.59 pcs	Cadenza
50	NKS	*QNks-AxC.ipbb-5B*	KB13, KB14	5B	195.5–217.6	658739979-588645321	3.6	9	1.72 pcs	Avalon
51	NKS	*QNks-AxC.ipbb-6A*	KA14/KO14/ KB13,14	6A	79.0–133.9	388058969-595563998	7.2	22	3.83 pcs −3.15 pcs	Avalon Cadenza
52	NKS	*QNks-AxC.ipbb-7B*	KA13/KO13	7B	67.2–70.5	733490728-741395913	3.6	12	−1.22 pcs 2.35 pcs	Cadenza Avalon
53	NKM	*QNkm-AxC.ipbb-1D*	KO14	1D	105.2–125.0	494063266-556487416	4.0	16	−8.03 pcs	Cadenza
54	NKM	*QNkm-AxC.ipbb-2A*	KA14	2A	184.6–211.9	738989334-761248549	3.9	25	−3.00 pcs	Cadenza
55	NKM	*QNkm-AxC.ipbb-2D.1*	KB14, KB15	2D	13.4–35.8	13677182-27925883	4.0	15	−2.98 pcs	Cadenza
56	NKM	*QNkm-AxC.ipbb-2D.2*	KO14, KO15, Koav	2D	69.6–106.8	650956549-70951376	4.3	13	−3.79 pcs	Cadenza
57	NKM	*QNkm-AxC.ipbb-3A*	KO14	3A	0.0–11.1	1309010 -12997670	3.6	10	−6.03 pcs	Cadenza
58	NKM	*QNkm-AxC.ipbb-3B*	KB13, KB15, KBav	3B	166.7–208.0	756120911-794813268	4.4	14	−4.69 pcs	Cadenza
59	NKM	*QNkm-AxC.ipbb-4A*	KB14, KA15	4A	111.4–156.0	705723286-719260469	3.6	9	−3.59 pcs	Cadenza
60	NKM	*QNkm-AxC.ipbb-4B*	KB14, KA15	4B	98.2–111.0	653949465 -660466325	4.5	17	−1.81 pcs	Cadenza
61	NKM	*QNkm-AxC.ipbb-5A*	KB13, KB14, KB15, KBav, KA13/ KBav, KA14	5A	0.0–41.1	414167574-485201230	9.4	28	−4.87 pcs 3.23 pcs	Cadenza/ Avalon
62	NKM	*QNkm-AxC.ipbb-6B*	KA15	6B	17.9–38.7	22818712-182321331	4.1	14	−4.47 pcs	Cadenza
63	NKM	*QNkm-AxC.ipbb-7A*	KA13, KO15	7A	121.0–137.3	638166554-669729056	3.9	11	−5.18 pcs	Cadenza
64	TKW	*QTkw-AxC.ipbb-1D*	KB15	1D	61.1–79.3	435933385-462203545	4.1	13	−1.06 g	Cadenza
65	TKW	*QTkw-AxC.ipbb-3B*	KO14,KO15	3B	181.3–227.4	763896022-816628413	3.2	12	−1.12 g	Cadenza
66	TKW	*QTkw-AxC.ipbb-3D*	KA14, KO15	3D	0.0–22.3	29165565-64767582	4.1	13	−1.77 g	Cadenza
67	TKW	*QTkw-AxC.ipbb-4D*	KA13, KO13	4D	21.4–60.6	3612555-32347333	9.0	26	−2.1 g 0.96 g	Cadenza Avalon
68	TKW	*QTkw-AxC.ipbb-5A*	KA13,KO13,KO15,KOav	5A	0.0–22.4	414167574-459003112	7.7	25	−0.89 g	Cadenza
69	TKW	*QTkw-AxC.ipbb-5B*	KO15/KB15	5B	125.2–147.7	558119994-596438283	5.6	18	1.84 g −0.93 g	Avalon Cadenza
70	TKW	*QTkw-AxC.ipbb-5D*	KA14	5D	76.1–104.0	351397580-434543581	4.4	14	−1.64 g	Cadenza
71	TKW	*QTkw-AxC.ipbb-6A*	KO13,KOav	6A	51.3–64.3	21520673-51409030	7.1	18	1.21 g	Avalon
72	TKW	*QTkw-AxC.ipbb-7D*	KO13, KBav	7D	40.2–46.7	555058879	3.4	9	0.54 g	Avalon
73	YM2	*QYM2-AxC.ipbb-1B*	KA15	1B	129.9–159.7	655781604-670783705	3.8	14	76.6 g	Avalon
74	YM2	*QYM2-AxC.ipbb-1D*	KB13,KB14	1D	61.3–105.0	435933385-494063266	4.2	18	69.2 g	Avalon
75	YM2	*QYM2-AxC.ipbb-2D.1*	KA13,KA14, KO13,KOav	2D	35.5–72.7	30149107 -81836821	4.7	17	−10.8 g	Cadenza
76	YM2	*QYM2-AxC.ipbb-2D.2*	KB14,KB15, KO13,KO15	2D	87.2–192.6	450999021,0	6.3	38	−78.7 g	Cadenza
77	YM2	*QYM2-AxC.ipbb-3B*	KA15	3B	168.8–189.3	753668293-775561953	3.9	4	38.3 g	Avalon
78	YM2	*QYM2-AxC.ipbb-4D*	KO13,KOav	4D	62.9–94.8	398908263-455253024	4.6	18	−47.2 g	Cadenza
79	YM2	*QYM2-AxC.ipbb-5A.1*	KB13,KB15, KAav	5A	89.4–91.6	572350283-582961392	11.9	54	615.5 g	Avalon
80	YM2	*QYM2-AxC.ipbb-5A.2*	KA14, KO14	5A	175.6–202.6	706429491-680066867	5.4	18	10.32 g	Avalon
81	YM2	*QYM2-AxC.ipbb-5D*	KA14	5D	33.4–71.2	40183784-120614528	3.6	10	7.52 g	Avalon
82	YM2	*QYM2-AxC.ipbb-6A*	KA14	6A	30.2–54.2	13806421-31354988	4.7	15	11.1 g	Avalon
83	YM2	*QYM2-AxC.ipbb-7B*	KA13,14	7B	57.2–64.7	711362280-730115050	3.6	9	−7.84 g 6.85 g	Cadenza Avalon

The number of identified QTLs found in the data from the three different regions varied significantly, and most MTAs were found in the Northern Kazakhstan data (50 QTLs), followed by the Southern (45 QTLs) and then Central sites (37 QTLs), of Kazakhstan (Table 3). Despite that more QTLs were identified in the Northern station data, the number of associations for the PT and NKS was nearly twice as high as that found in the Central and Southern regions (Table 3).

### QTL mapping for traits related to plant adaptation in the Avalon × Cadenza DH population

A total of 18 QTLs was identified for plant adaptation-related traits, 12 of them detected in the PH trait (Table 4). The majority of QTLs were detected at the irrigated KO site (eight QTLs), while at the non-irrigated KB and KA sites, five QTLs were recorded at each location. Field trials in the three sites led to the identification of only four common QTLs (1D, 2D, 5B, and 6A), and one of them, *QHt-AxC.ippb-1D*, was identified over the years in KB sites. Another QTL for SMT, *QHt-AxC.ippb-2D*, was identified at the KO region, and Avalon was the donor of the increasing alleles. For SMT, we detected only two QTLs (1A), which were identified in the KB region, and in both cases, Cadenza was the donor of the increasing alleles (Table 4).

**Figure 3:**
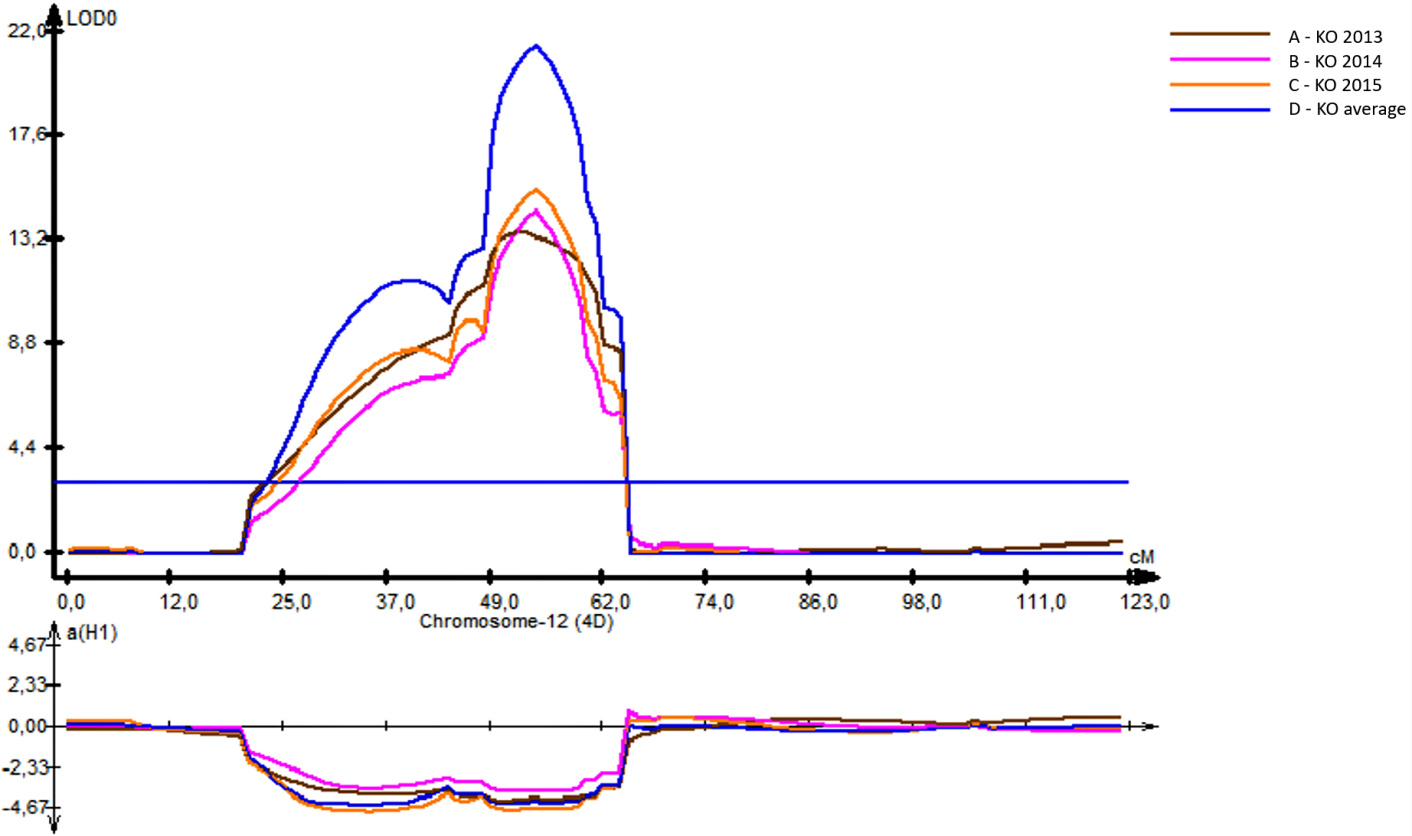
The position of identified quantitative trait locus (QTL) for plant height (PH) revealed on 4D chromosome using irrigated field trials at the Kyzylorda (KO, South Kazakhstan) in 2013–2014–2015. .

Unlike the HT and SMT analyses, where only a few QTLs were identified, twelve QTLs were genetically mapped for PH (Table 4). The R ^2^ for the PH ranged from 10% (for *QPh-AxC.ipbb-3A*) to 48% (*QPh-AxC.ipbb-4D*) (Table 4), where the latter QTL was mapped in the vicinity of the *Rht1* gene. *QPh-AxC.ipbb-4D* had the highest LOD score (21.5) compared to the other PH-associated QTLs; however, this QTL was significant only at the KO irrigated site (Table 4, Fig. 3). At the Northern KB site, five QTLs on chromosomes 2A, 2B, and 5A were identified. For those five QTLs, four alleles for increasing height were from Avalon, and only the QTL on 5A (135.2–183.7 cM) had the increasing height allele from Cadenza (Table 4).

### QTL mapping for yield components in the Avalon × Cadenza DH population

A total of 65 stable QTLs were identified for six traits directly related to grain yield. The number of stable QTLs per trait is ranged from 9 in TKW to 12 in SL and NKM. Three QTLs for NKS (Avalon), TKW (Cadenza/Avalon), and YM2 (Cadenza) were mapped on chromosome 4D in the vicinity of the *RhtMrkD1* gene associated with reduced plant height. The largest number of QTLs were identified at the KB site (39 QTLs) followed by KO (33 QTLs) and KA (31 QTLs) (Table 3). Twelve QTLs were identified for SL, and their R ^2^ ranged from 11% (*QSl-AxC.ipbb-6A)* to 53% (*QSl-AxC.ipbb-2D.1*). The locus *QSl-AxC.ipbb-2D.1* was detected at both the KB and KO sites. However, the largest QTL effect for SL was *QSl-AxC.ipbb-5A.2* (0.85) , with Avalon being the donor of the favorable allele (Table 4, Table S2, and Fig. S2).

**Figure 4:**
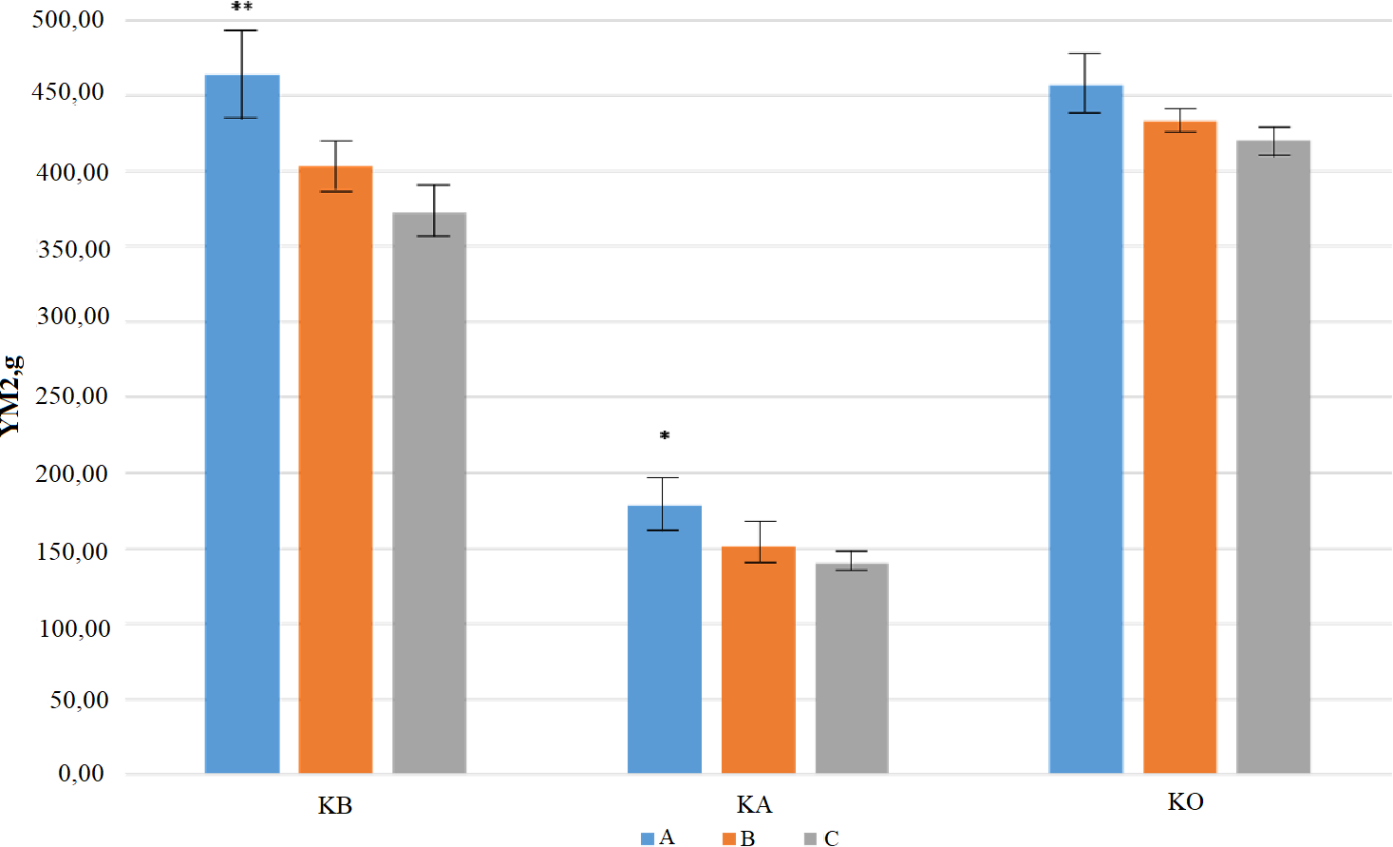
A comparative effect of groups with a different number of positive quantitative trait loci (QTL) for the number of kernels per spike (NKS) on average yield performance in three studied regions. **(**A) represents the group with high, (B) with middle, and (C) with low number of SNPs with favorable alleles for each identified QTL for NKS (based on data in Table 4). KB, KA, and KO are three tested sites in Northern, Central, and Southern Kazakhstan, respectively. YM2 is the yield per square meter. ** - *P* < 0.01, and * - *P* < 0.05.

Eleven QTLs were identified for PT, where the R^2^ values were ranged from 17% (for *QPt-AxC.ipbb-1A*) to 66% (for *QPt-AxC.ipbb-2D*). Three QTLs (*QPt-AxC.ipbb-3D.1, QPt-AxC.ipbb-3D.2,* and *QPt-AxC.ipbb-3D.3)* were genetically mapped to different regions of chromosome 3D by using trial data from the KA and KB sites (Table 4). Of the eleven QTLs for NKS, the largest QTL effects were due to *QNks-AxC.ipbb-6A* (3.83) and *QNks-AxC.ipbb-7B* (2.35), and in both cases, Avalon was the donor of these alleles associated with an increasing effect. The largest number of QTLs for NKS was identified in the data from KB (nine QTLs), where three QTLs were located in each of the A and D genomes, and four QTLs were in the B genome (Table 4). The 101 studied DH lines were separated into groups with high (from 6 to 9), middle (from 4 to 5), and low (from 1 to 3) numbers of positive QTLs (favorable alleles of significant SNP), and groups was represented by 19, 41, and 41 accessions, respectively. The unpaired *t*-test for DH lines harvested in Northern Kazakhstan suggested that the YM2 of the group with the high number of positive QTLs for NKS was significantly superior in comparison to the middle (*P* < 0.05) and low (*P* < 0.01) groups (Fig. 4). A similar outcome was recorded in Central Kazakhstan, where only three QTLs for NKS were identified, and samples with three positive QTLs (*n* = 8 samples) were having significantly higher YM2 (*P* < 0.05) in comparison to the group of DH lines with none or one positive QTL (*n* = 63 samples). A different result was recorded in Southern Kazakhstan, as the groups with more positive QTLs for NKS showed no statistical advantages in averaged YM2 over the groups with less positive QTLs (Fig. 4, Table S3).

Of the twelve QTLs for NKM, the largest QTL effects were observed for *QNkm-AxC.ipbb-1D* (−8.03) and *QNkm-AxC.ipbb-3A* (−6.03), and in both cases, Cadenza was the donor of these alleles associated with an increasing effect. The R^2^ values for nine QTLs for TKW ranged from 9% (for *QTkw-AxC.ipbb-7D*) to 26% (for *QTkw-AxC.ipbb-4D*)*.* The QTL with the largest LOD score (9.0) for TKW was *QTkw-AxC.ipbb-4D*. However, the most important QTL with the largest effect (1.84 g, Avalon) was *QTkw-AxC.ipbb-5B*, where the allele associated with an increasing effect came from Avalon (Table 5). Finally, eleven QTLs were identified for YM2, including *QYM2-AxC.ipbb-5A.1*, which was the locus with the largest LOD score (11.9) and QTL effect (615.5) at the KB/KA sites (Table 4, Table S2 , Fig. S2).

**Table 5:** List of identified QTLs based on the field trials of the A × C doubled haploid mapping population and comparative analyses with the associations revealed in previously published reports.

No	**QTL**	**Chr**	**Position, cM**	**Position chr., cM, markers, region**	**References**
*1*	*QHt-AxC.ippb-6A*	6A	30.3–92.3	99.39 (KO 2013)	Turuspekov et al. (2017)
*2*	*QPh-AxC.ipbb-2A*	2A	72.0–107.6	70 (xgwm359)	Griffiths et al. (2012)
*3*	*QPh-AxC.ipbb-2D*	2D	13.1–51.3	32 (xgwm261)	Griffiths et al. (2012)
				37	Ma et al. (2015)
				28.18 (KO2015)	Turuspekov et al. (2017)
*4*	*QPh-AxC.ipbb-3A*	3A	49.4–98.5	77	Ma et al. (2015)
*5*	*QPh-AxC.ipbb-3B*	3B	62.7–116.4	85 (xgwm285)	Griffiths et al. (2012)
				59.17(KA2014, KO2014)	Turuspekov et al. (2017)
*6*	*QPh-AxC.ipbb-4D*	4D	20.3–65.1	1 (RhtMrkD1)	Griffiths et al. (2012)
				48	Ma et al. (2015)
*7*	*QPh-AxC.ipbb-5A.1*	5A	8.3–40.4	41.7–46.5	Abugalieva (2007)
				1 (xgwm293)	Griffiths et al. (2012)
*8*	*QPh-AxC.ipbb-5A.2*	5A	50.5–81.5	60.4	Griffiths et al. (2012)
				87	Ma et al. (2015)
*9*	*QPh-AxC.ipbb-5B*	5B	42.7–133.2	65 (xgwm 408)	Griffiths et al. (2012)
*10*	*QPh-AxC.ipbb-6B*	6B	111.1–136.0	134	Ma et al. (2015)
*11*	*QSl-AxC.ipbb-1B*	1B	7.2–47.1	30.1–44.2	Jantasuriyarat et al. (2004)
*12*	*QSl-AxC.ipbb-2A*	2A	93.4–107.3	92.1	Onyemaobi et al. (2018)
*13*	*QSl-AxC.ipbb-2D.1*	2D	24.1–51.0	28.5	Zhou et al. (2017)
				28.18 (KA2014)	Turuspekov et al. (2017)
*14*	*QSl-AxC.ipbb-2D.2*	2D	86.9–193.3	62.6–93.9	Echeverry-Solarte et al. (2015)
*15*	*QSl-AxC.ipbb-3D*	3D	40.2–98.2	76.1–133.2	Abugalieva (2007)
*16*	*QSl-AxC.ipbb-4A*	4A	104.7–121.6	88.1–109.5	Jantasuriyarat et al. (2004)
*17*	*QSl-AxC.ipbb-5A.1*	5A	35.3–63.5	78.9	Zhou et al. (2017)
				77.7	Onyemaobi et al. (2018)
*18*	*QSl-AxC.ipbb-5A.2*	5A	91.6–123.7	82.0–100.8	Abugalieva (2007)
				84.2	Onyemaobi et al. (2018)
*19*	*QSl-AxC.ipbb-6A*	6A	64.1–101.2	88.2	Zhou et al. (2017)
*20*	*QPt-AxC.ipbb-1A*	1A	39.2–55.4	1AS (30)	Quarrie et al. (2005)
				52.1–60.0	Abugalieva (2007)
				45.65 (KA2013)	Turuspekov et al. (2017)
*21*	*QPt-AxC.ipbb-2B*	2B	79.0–87.2	60.89 (KA2013)	Turuspekov et al. (2017)
*22*	*QPt-AxC.ipbb-4B*	4B	98.3–109.3	4BL/S (62.8–90.7)	Quarrie et al. (2005)
*23*	*QPt-AxC.ipbb-5A*	5A	98.1–105.9	5AL (107.6, 108.7)	Quarrie et al. (2005)
*24*	*QPt-AxC.ipbb-5D*	5D	4.3–16.3	9.0–14.4	Abugalieva (2007)
*25*	*QNks-AxC.ipbb-1A*	1A	16.3–31.2	1AS (30)	Quarrie et al. (2005)
*26*	*QNks-AxC.ipbb-4B*	4B	47.5–62.3	4BL (62.8–90.7)	Quarrie et al. (2005)
*27*	*QNkm-AxC.ipbb-2D.1*	2D	13.4–35.8	33	Ma et al. (2015)
*28*	*QNkm-AxC.ipbb-5A*	5A	0.0–41.1	12	Ma et al. (2015)
*29*	*QTkw-AxC.ipbb-3D*	3D	0.0–22.3	0–7.4	Tura et al. (2020)
*30*	*QTkw-AxC.ipbb-4D*	4D	21.4–60.6	4DL (22.6)	Quarrie et al. (2005)
				25	Ma et al. (2015)
				35.2–35.8	Tura et al. (2020)
*31*	*QTkw-AxC.ipbb-5A*	5A	0.0–22.4	5	Ma et al. (2015)
*32*	*QTkw-AxC.ipbb-5B*	5B	125.2–147.7	144.1 (KO2015)	Turuspekov et al. (2017)
				149.9–161.5	Tura et al. (2020)
*33*	*QTkw-AxC.ipbb-5D*	5D	76.1–104.0	5DL (61.1)	Quarrie et al. (2005)
				94–96.2	Tura et al. (2020)
*34*	*QTkw-AxC.ipbb-6A*	6A	51.3–64.3	65	Ma et al. (2015)
				58.2–66.1	Tura et al. (2020)
*35*	*QYM2-AxC.ipbb-1B*	1B	129.9–159.7	105-110	*Tura et al., (2020)*
*36*	*QYM2-AxC.ipbb-2D.1*	2D	35.5–72.7	36	*Ma et al., (2015)*
				52.3	El-Feki et al. (2018)
*37*	*QYM2-AxC.ipbb-2D.2*	2D	87.2–192.6	94.63	Lopes et al. (2015)
				76.2–76.3	Tura et al. (2020)
*38*	*QYM2-AxC.ipbb-3B*	3B	168.8–189.3	188	Ma et al. (2015)
*39*	*QYM2-AxC.ipbb-5D*	5D	33.4–71.2	21.60 (KA14)	Turuspekov et al. (2017)
*40*	*QYM2-AxC.ipbb-7B*	7B	57.2–64.7	66.45 (KA2014)	Turuspekov et al. (2017)

## Discussion

### Yield assessment of the A × C DH population in three contrasting regions of Kazakhstan during the period 2013–2015

The field performance of the studied population significantly depended on geographic locations and key environmental parameters, including mean temperature, average rainfall, day length, soil quality, and etc. (Table 1). Therefore, these factors, particularly temperature and amount of precipitation in key stages of plant growth, may lead to different plant performances of the same collection of samples in different wheat-growing regions (Two-factorial ANOVA in Raw meteorological data file). In the present study, the correlation analysis showed negative influence of late heading time on major yield components-YM2 and TKW in Central and South regions, but not in the North (Fig. 1). Traditionally, the requirements for the early development of wheat in the Northern region were negated by the fact that local breeders were mostly focusing on grain quality parameters and, therefore, they targeted lines with an early flowering time (*Kamran, Iqbal & Spaner, 2014; Tshikunde et al., 2019*) and late SMT. However, the analysis of meteorological data revealed that heavy rains in early September were occurring more often in Northern Kazakhstan than in previous decades, which might result in a change in the breeding goals toward an early SMT as well. Hence, the negative correlation of yield-related traits with HT and SMT observed in this study, although not significant, is additional evidence of the necessity to adjust the local breeding priorities in northern parts of Kazakhstan.

The analysis of the averaged YM2 revealed 9, 22, and 26 DH lines that exceeded the YM2 of the local standard cultivars in the Northern, Central, and Southern regions, respectively. In addition, 86, 24, and 3 DH lines exceeded the YM2 of the Cadenza (parent) in the KA, KB and KO, respectively. Two particular lines, A × C52 and A × C55, demonstrated adaptability to both non-irrigated and irrigated sites and showed higher productivity than the local standards in all three of the studied regions (Fig. 2). The application of a GGE biplot analysis suggested some more insights into the assignment of particular DH lines for their possible usage in breeding projects at the three different regions using YM2. This result is particularly important as the correlation test suggested that average YM2 in the non-irrigated sites KB and KA were highly correlated (*P* < 0.01), and the yield in both locations was not correlated with the irrigated site in KO (*P* < 0.811).

### Comparative analysis with associations revealed in previously published reports

The QTL analyses in the three regions led to the identification of 83 stable QTLs that were significant for nine agronomic traits in two and more environments (Table 4). Notably, the least number of associations was identified for HT and SMT, which is an indication of a narrow range of heading times in the population tested under these new environments. On the other hand, the determination of only a few QTLs associated with HT and SMT suggests that the majority of those identified for yield-related traits were not associated with the pleiotropic effects of major genes.

A comparison of the mapped QTLs analyzed in this study with those from other previous studies indicated that 40 QTLs matched known associations (Table 5). Twelve associations matched the results from studies of the SQ1 × CS DH mapping population (*Quarrie et al., 2005; Abugalieva, 2007*), where five associations with PT, and two with SL, NKS, and TKW, were identified through studies in Southeastern Kazakhstan. Another nine associations were identical to the genetic positions of QTLs identified with the analyses of six traits using GWAS based on the assessment of common wheat in three different regions of Kazakhstan (*Turuspekov et al., 2017*). Notably, five of those nine associations were also genetically mapped in other GWAS conducted around the world.

The literature survey demonstrated that 16 out of the 84 QTLs identified in our study had also been detected in previous QTL mapping studies for PH, NKM, TKW, and YM2 traits using the A ×C population in Europe (*Griffiths et al., 2012; Ma et al., 2015*). The majority of those matches were found for PH (nine QTLs), followed by TKW (three QTLs), NKS, and YM2 with two QTLs for each trait (*Ma et al., 2015*) (Table 5).

### Assessment of presumed novel QTLs based on the field trials of the A ×C DH population

The identification of 43 novel putative QTLs identified in this work underlines the importance of collaborative efforts as the A ×C was developed as a reference DH population within the UK Wheat Genetic Improvement Network (http://www.wgin.org.uk). These results are additional evidence of the importance of extensive germplasm exchange. On the other hand, the identification of new highly significant MTAs underlies the significance of field trials under diverse environmental niches, particularly in those countries where cultivation plays an enormous role in global food security. Hence, the combination of these two factors may lead to the discovery of new important MTAs controlling both plant adaptation-related traits and yield-related traits.

For instance, CIM allowed for the identification of seven novel putative QTLs for PH, including four associations revealed in the Northern region of Kazakhstan (Table 4). One of those QTLs, *QPh-AxC.ipbb-5A.1*, possibly affects both NKS and TKW in the Northern region as their mapping intervals on chromosome 5A co-localized (Table 4). Similar findings were found from studies in the Southern region as *QPh-AxC.ipbb-3D* and *QPh-AxC.ipbb-4D* share locations with QTLs for NKS and TKW, respectively (Table  4). In the search for novel QTLs in TKW, three out of five QTLs were revealed in the Northern region (Table 4). Notably, *QTkw-AxC.ipbb-1D* (Cadenza) had a matching QTL position with the association for YM2 (*QYM2-AxC.ipbb-1D*, (Avalon)) in Northern Kazakhstan, and *QTkw-AxC.ipbb-3B* (Cadenza) and *QTkw-AxC.ipbb-6A* (Avalon) matched corresponding QTLs for YM2 in Southern Kazakhstan. As seen from Fig. 1, NKS was a highly significant trait for yield performance in Northern Kazakhstan; therefore, it was important to assess whether the identified QTLs for NKS have contributed to the average YM2 over three years. Hence, the DH mapping population was partitioned into groups with the high, middle, and low number of QTLs that carry SNPs with favorable alleles. The evaluation of average YM2 in those groups has demonstrated that having more positive QTLs is highly advantageous over lines with a low number of positive QTLs for plant performances (Fig. 4). Therefore, the study is another confirmation that the accumulation of favorable QTLs is a promising approach in wheat breeding conducted in specific environments (*Würschum, Leiser & Langer, 2018; Tshikunde et al., 2019*). Still, the results suggest that the higher number of favorable QTLs for NKS does not always seem significant for increased yield, as it was notable for KO (South Kazakhstan) site (Fig. 4). Evidently, despite the benefit of having more positive QTLs for NKS in non-irrigated KB and KA sites, the irrigated KO condition have some masking effect on this advantage for yield performance.

## Conclusions

The field assessment of 101 A × C DH spring lines in three different regions of Kazakhstan revealed phenotypic variation in nine agronomic traits. The correlational analysis suggested that early HT and SMT in the Southern and Central regions were important for higher grain yield and, therefore, the identified favorable correlations were negative. In the Northern region, where Kazakhstan had more than 80% of the area under wheat, the correlation was not significant, although it was also negative. Traditionally, spring wheat in this region was bred for higher grain quality at the expense of yield productivity. The comparative assessment of DH lines with local standard cultivars in the three regions revealed that 9, 22, and 26 lines were superior to their corresponding standards in the Northern, Central, and Southern regions, respectively. Two lines, A ×C52 and A × C55, demonstrated broad adaptability and showed higher productivity than the local controls in all three regions. Thus, all these identified lines can be successfully introduced into regional breeding projects targeting higher grain yield. The analysis of the A × C DH mapping population allowed for the detection of 232 QTLs for nine agronomic traits. The comparative evaluation of the total number of QTLs suggested that 83 QTLs were significant in two and more environments and were considered as stable QTLs. A literature survey showed that 40 out of the 83 QTLs had been previously reported, suggesting that these results are robust, and that 43 QTLs identified in this study are presumably novel. The comparative study of DH lines in Northern and Central Kazakhstan with the high, middle, and low number of QTLs for NKS with favorable alleles of significant SNPs has clearly indicated that lines with higher accumulation of positive QTLs have significantly higher grain yield. Identified QTLs could be used in local breeding activities for marker-assisted selection to obtain a higher yield performance and, hence, contribute to the improvement of the total wheat productivity in the country.

##  Supplemental Information

10.7717/peerj.10733/supp-1 Supplemental Information 1 Genotype file of doubled haploid mapping population Avalonx Cadenza to consist of 3,647 polymorphic DNA markers

10.7717/peerj.10733/supp-2 Supplemental Information 2 List of stable QTLs identified in the Avalon x Cadenza mapping population

10.7717/peerj.10733/supp-3 Supplemental Information 3 A comparative effect of groups with different number of positive quantitative trait loci (QTL) for the number of kernels per spike on the performance of yield (YM2) in three regions of Kazakhstan. The grouping of positive QTLs for NKS with average yield per square meter (YM2) in KB, KA, and KO experimental sites in Northern, Central, and Southern Kazakhstan, respectively. Average YM2 is calculated over three studied years (2013–2015).

10.7717/peerj.10733/supp-4 Supplemental Information 4 The locations of three experimental sites in Kazakhstan

10.7717/peerj.10733/supp-5 Supplemental Information 5 Genetic map of QTLs associated with plant adaptation and yield components identified the Avalon x Cadenza DH population The markers names are shown on the right and positions of marker loci are shown on the left of the linkage maps in centimorgans (cM). Significant markers, the identified QTLs, blue for traits related to yield component QTLs, green for traits related to plant adaptation QTLs, and pink for genes.

10.7717/peerj.10733/supp-6 Supplemental Information 6 Raw field data from three regions of Kazakhstan

10.7717/peerj.10733/supp-7 Supplemental Information 7 Raw meteorological data for three experimental sites in 2013–2020 years

## Additional Information and Declarations

### Data Availability

The following information was supplied regarding data availability: The raw data are available in the Supplementary Files.
