# Supplementary figures and images for: QTL mapping of agronomic traits in wheat using the UK Avalon × Cadenza reference mapping population grown in Kazakhstan
Source: PeerJ. 2021 Feb 18;9:e10733. doi: 10.7717/peerj.10733 (PMC7897413; doi:10.7717/peerj.10733)

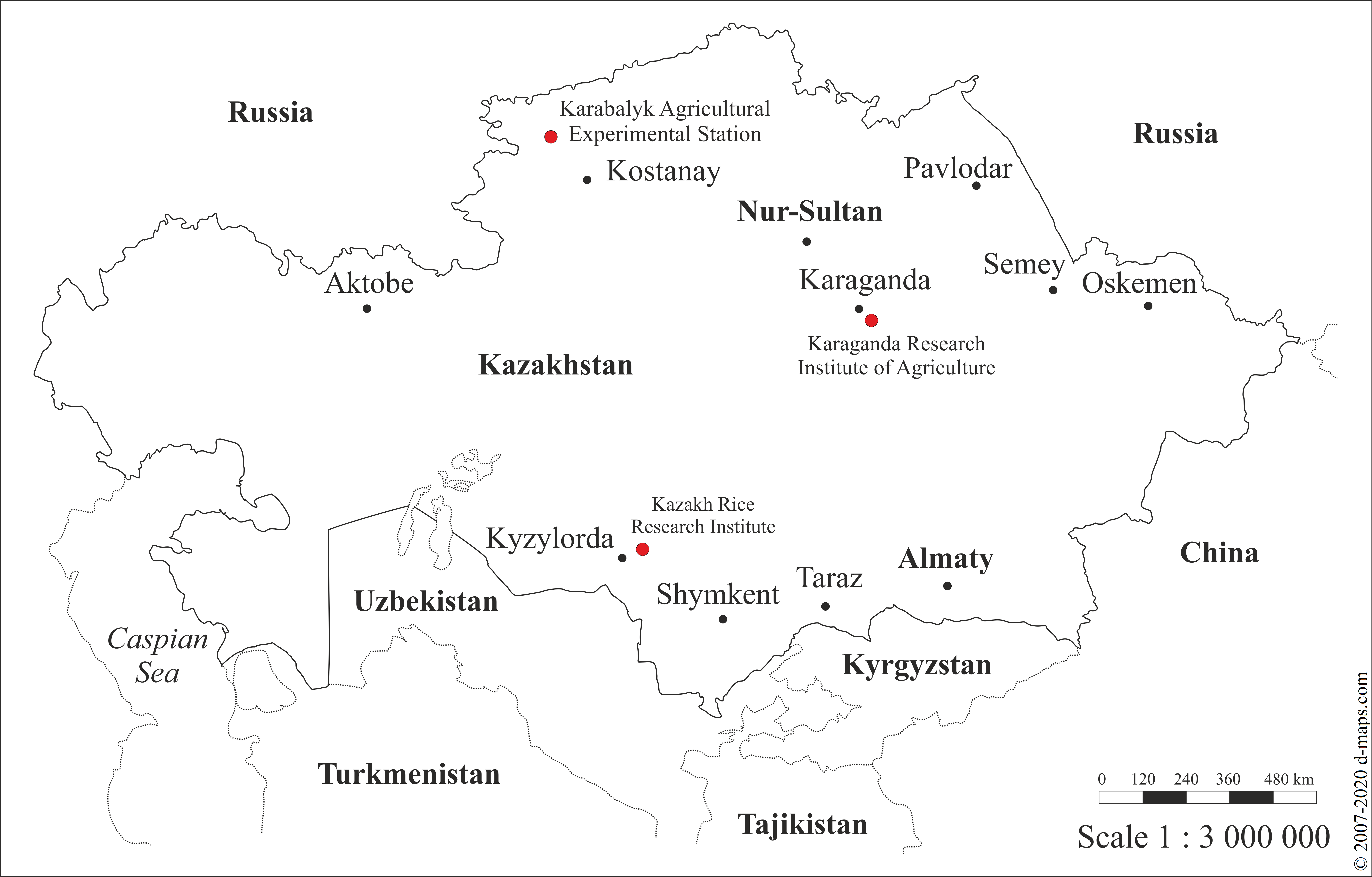

Supplement: Supplemental Information 4 [file peerj-09-10733-s004.png]
